# A Systematic Review and Meta-Analysis of Percutaneous Coronary Intervention (PCI) and Coronary Bypass Grafting (CABG) Outcomes in Indigenous vs. Non-Indigenous Australians

**DOI:** 10.7759/cureus.58172

**Published:** 2024-04-13

**Authors:** Moeed Ali Karim

**Affiliations:** 1 Cardiology, University of Sydney, Sydney, AUS

**Keywords:** major adverse cardiac effect (mace), systematic review and meta analysis, indigenous, coronary artery bypass grafting(cabg), primary pci

## Abstract

Introduction: A major cause of death, coronary artery disease (CAD) often necessitates invasive procedures like coronary bypass grafting (CABG) and percutaneous coronary intervention (PCI). Cardiovascular outcomes vary between indigenous and non-indigenous Australian people; however, comprehensive knowledge of these differences is absent.

Methodology: To compare PCI and CABG results between indigenous and non-indigenous Australians, a systematic review and meta-analysis were carried out. Included were 10 retrospective observational studies that examined mortality, cardiovascular events, comorbidities, and operative success rates. Databases spanning 2014 to 2024 were searched, and research that directly compared Australia's indigenous and non-indigenous populations was among the inclusion criteria.

Results: Within 30 days of surgery, indigenous Australians receiving PCI had greater rates of comorbidities and were at higher risk of long-term mortality and MACE. Similarly, there was a greater long-term death rate among indigenous patients following CABG. Cultural safety, socioeconomic factors, and regional factors affecting treatment delays and access to care all affected disparities. For 30-day mortality, the pooled analysis shows an odds ratio of 1.04 (95% CI 0.78, 1.40), indicating no meaningful difference. The total odds ratio for unfavorable occurrences is 1.07 (95% CI 0.86, 1.33), meaning there is no statistically significant difference between Indigenous groups and those that are not.

Conclusion: Indigenous Australians continue to have worse cardiovascular outcomes after PCI and CABG procedures, even with similar procedural success rates. To ensure equitable cardiovascular outcomes for indigenous groups, targeted therapies targeting underlying risk factors, increased access to culturally appropriate care, and decreased obstacles to healthcare access are critical.

## Introduction and background

One of the major causes of mortality is coronary artery disease or CAD. The non-surgical, invasive percutaneous coronary intervention (PCI) method is used to increase blood flow to the ischemic region by relieving coronary artery constriction or blockage [1]. Usually, many techniques are used to do this, the most popular being inflating the narrow part or inserting a stent to keep the artery open. Further treatment involves the use of harvested venous or arterial conduits to bypass atheromatous obstructions in a patient's coronary arteries. This significant surgical procedure is known as coronary artery bypass grafting, or CABG [2]. By restoring blood flow, the bypass helps to alleviate anginal symptoms and restore function and viability to the ischemic myocardium. The most frequent major surgical surgery, over 400,000 CABG procedures, is carried out per year; however, as the utilization of other treatments, such as medicinal therapy and percutaneous coronary intervention (PCI), has grown, surgical trends have fallen [3].

Significant progress has been made in the last 30 years in the treatment of cardiovascular disease, leading to better results on a worldwide scale. However, there are still differences in health across different demographic groups, and these advancements in treatment and results have not always been spread fairly. There is still a significant difference in life expectancy between indigenous and non-indigenous Australians in Australia, most of which is caused by cardiovascular disease [4]. The frequency of acute coronary syndromes and ischemic heart disease varies significantly across indigenous Australian communities, which is a significant contributing factor to premature death [5]. Over the last 15-20 years, there have been encouraging improvements in the health outcomes for indigenous Australians. Since 1998, the number of Indigenous Australians who die from heart-related illnesses has almost decreased by half. There has also been an improvement in the availability of prompt diagnosis and guidelines-based treatments for heart disease [6].

Notwithstanding, some obstacles persist, such as unequal opportunities for angiography and revascularization among indigenous Australians, and an increasing disparity has been seen in certain metrics, such as hospital admission rates for cardiovascular conditions [7]. Furthermore, there is a dearth of information about the characteristics and results of indigenous Australians undergoing percutaneous coronary intervention (PCI), the most popular method of revascularization. Finding any variations in PCI cohorts and possible reasons for these variations might point to care systems that need to be addressed, considering the disparities in the outcomes of cardiovascular disease. The purpose of this study was to compare the patient characteristics, presentations, and outcomes of indigenous and non-indigenous Australians undergoing PCI in metropolitan and larger regional centers in Victoria, Australia. Victoria is a state in the southeast of the nation with a population of about 6.4 million people, or 7.2% of Australia's indigenous population [8]. There is a notable dearth of thorough data comparing the results of percutaneous coronary intervention (PCI) and coronary artery bypass grafting (CABG) between indigenous and non-indigenous populations in Australia, despite the significant burden of cardiovascular disease (CVD) among indigenous Australians. Prior research has mostly looked at overall cardiovascular outcomes in Native American communities or has looked at the results of PCI and CABG independently [9]. So far, there hasn't been a systematic review and meta-analysis that compares the efficacy and results of various revascularization operations between indigenous and non-indigenous Australians.

This information gap emphasizes how crucial it is to carry out a systematic review and meta-analysis to compile the existing data and provide a thorough grasp of the relative efficacy of PCI and CABG in indigenous communities. This study intends to clarify any differences between indigenous and non-indigenous Australians undergoing PCI and CABG in terms of procedural success rates, long-term mortality, cardiovascular events, complications, and health-related quality of life outcomes by methodically analyzing the body of existing literature.

## Review

Methodology

Search Strategy

To evaluate the results of coronary artery bypass grafting (CABG) and percutaneous coronary intervention (PCI) in indigenous and non-indigenous Australians, we carried out a systematic review and meta-analysis. From the last decade (2014-2024), a thorough search was conducted across several electronic databases, including PubMed and Google Scholar. Refinement of search queries was achieved by combining Medical Subject Headings (MeSH) terms with pertinent keywords, such as "percutaneous coronary intervention," "coronary artery bypass grafting," "Indigenous," "Aboriginal Australians," and "Torres Strait Islanders," among others, with Boolean operators.

Inclusion Criteria

The inclusion criteria included studies comparing PCI and CABG outcomes between indigenous and non-indigenous Australians, studies reporting primary outcomes such as procedural success rates, mortality, cardiovascular events, complications, and health-related quality of life, as well as randomized controlled trials, cohort studies, and observational studies.

Exclusion Criteria

The exclusion criteria included studies not conducted in Australia and studies not reporting outcomes of interest or not directly comparing indigenous and non-indigenous populations, as well as review articles, commentaries, editorials, and conference abstracts.

Data Extraction

Data extraction involved the independent screening of titles and abstracts by two reviewers to assess eligibility, followed by a full-text assessment of potentially eligible studies. Data were extracted using a standardized form capturing study characteristics, participant demographics, intervention details, and outcome measures as defined by the primary outcomes. Any discrepancies were resolved through consensus or consultation with a third reviewer.

Quality Assessment

The methodological quality of the included studies was assessed using the Newcastle-Ottawa Scale for observational studies. The data were analyzed by Review Manager Software 5.3. The effect size for continuous data is expressed as the mean difference and the 95% CI. The effect size for dichotomous data is expressed as the OR and the 95% CI. The χ2 test P-value and the I 2 value were used to determine the level of heterogeneity.

Results

**Figure 1:**
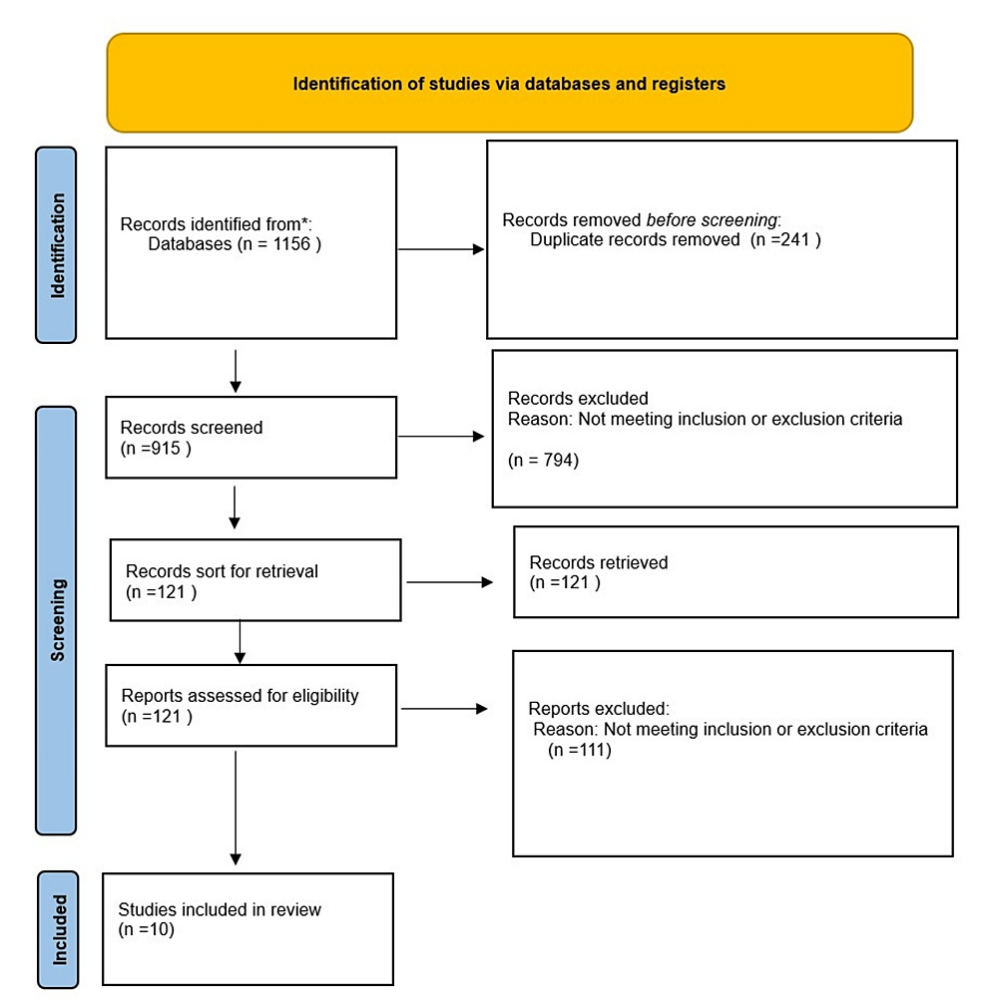
Identification of studies via databases and registers

In the process of the preliminary literature search, 1156 publications in all were found. After abstracts and titles were carefully evaluated, 121 publications were deemed relevant, and their complete texts were obtained for further analysis. Studies that did not explicitly examine the effects of PCI and CABG on Australians, both indigenous and non-indigenous, or that did not fit the inclusion requirements were subsequently eliminated. Following a stringent screening procedure, 10 studies were found to be appropriate for incorporation into the systematic review and meta-analysis. A Prisma flow diagram is seen in Figure 1.

The systematic review encompassed 10 retrospective observational studies investigating the outcomes of percutaneous coronary intervention (PCI) and coronary artery bypass grafting (CABG) procedures in indigenous Australians compared to non-indigenous Australians. These studies were conducted across diverse regions of Australia, with varying sample sizes and surgical interventions examined. Population sizes ranged widely, from as large as 41,146 individuals to as low as 72 participants, reflecting the breadth of research in this area. Surgical interventions included PCI, isolated CABG, and broader cardiac surgery procedures, providing a comprehensive overview of cardiac interventions among Indigenous populations.

Across the studies, indigenous patients consistently exhibited distinct demographic and clinical characteristics compared to their non-Indigenous counterparts. They were typically younger, more frequently female, and presented with a higher burden of comorbidities, highlighting potential disparities in health outcomes and healthcare access. Furthermore, indigenous patients experienced higher rates of adverse outcomes following cardiac interventions, including increased long-term mortality, 30-day mortality, major adverse cardiac events (MACE), and in-hospital bleeding incidents (Table 1).

**Table 1:** Characteristics of the studies reviewed MACE: Major Adverse Cardiovascular Events, PCI: Percutaneous Intervention, ATSI: Aboriginal and Torres Strait Islander, CABG: Coronary Artery Bypass Graft

Author	Study Type	Population Type	Population Number	Surgical Intervention	Results	Conclusion
L. Dawson et al. [5]	Retrospective observational	Indigenous Australians in Victoria	179	PCI	In multivariable analysis, Indigenous Australians had increased risks of long-term death, 30-day MACE, and in-hospital bleeding (HR 2.85, 95% CI 2.05–3.96). After controlling for age, sex, and comorbidities, 30-day mortality increased.	Indigenous Australians are more likely to have PCI complications. PCI results and clinical engagement need improvement.
Luke P Dawson et al. [7]	Multicentre, prospective observational	Indigenous and non-Indigenous Australians	41,146	PCI	Indigenous patients lived more in remote and impoverished locations. In multivariable analysis, Indigenous status was linked to higher risk of long-term mortality (HR 2.49, 95% CI 1.79–3.48; p < 0.0001), 30-day mortality (OR 2.78, 95% CI 1.09–7.12; p = 0.033), and 30-day MACE (OR 1.87, 95% CI 1.03–3.39; p =	Culturally responsive treatment approaches are needed for Indigenous Australians having PCI in urban and rural locations, who have higher death and adverse cardiac event rates.
H. Su et al. [8]	Retrospective observational	Aboriginal and Torres Strait Islanders (ATSI)	4489	PCI, Cardiac Surgery	Younger, more female ATSI patients had different angiography indications than non-ATSI patients. Similar PCI or surgery rates (35.6% vs 38.5%, p = 0.17), but ATSI patients had increased long-term mortality after adjusting for baseline variables (HR 1.80, 95% CI 1.42–2.27; p < 0.001).	Indigenous patients were more likely to have PCI and heart surgery, with comparable short-term outcomes but almost twice long-term mortality.
O'Brien et al. [10]	Retrospective observational	Indigenous vs. non-indigenous	Indigenous: 778	Isolated CABG	Patients from indigenous cultures tended to be younger, had more comorbidities, and were more likely to be women. Higher incidences of postoperative hemorrhage were seen (p = 0.001). There was comparable short-term mortality.	Despite variations in baseline characteristics, the short-term results after CABG for Indigenous Australians were not poorer. More research is required for long-term results.
Jessy A Nellipudi et al. [11]	Retrospective observational	Indigenous vs. non-indigenous	Indigenous: 1431	Isolated CABG	Indigenous individuals had greater comorbidities and presented at a younger age. Propensity-adjusted all-cause long-term mortality was more likely to occur in them (HR = 1.712, 95% CI: 1.288-2.277, p < 0.001).	Patients from Indigenous communities who had CABG had a greater chance of dying over time. Personalized preventative techniques might enhance post-CABG health outcomes.
Sarah Page et al. [12]	Retrospective observational	Indigenous vs. non-indigenous	Indigenous: 19.1%	Chest Pain in ED	Indigenous patients presented younger, had greater rates of CABG, and more cardiovascular risk factors. Major adverse cardiac events were much more common in them (AOR = 2.0, 95% CI [1.1, 3.8], p = 0.03).	In order to enhance the cardiovascular health outcomes for Indigenous individuals who arrive with chest discomfort, collaboration is required.
Kyi T.H. Win et al. [13]	Retrospective observational	Indigenous vs. non-indigenous	Indigenous: 72	Cardiac Surgery	Indigenous dialysis patients had higher comorbidities and seemed younger. Although the mortality rates were similar, there was a greater return to theatre rate (43% vs. 17%) among Indigenous patients.	The death rates of Indigenous patients having heart surgery were similar, but their return-to-the-theater rates were greater.
Edward R. Justo et al. [14]	Retrospective observational	Indigenous vs. non-indigenous	Indigenous: 123	Cardiac Surgery	The socioeconomic deprivation and complexity ratings of Indigenous patients were greater. The indigenous group had a worse 6-year survival rate (8.1% vs. 5.0%; HR = 2.1; 95% CI: 1.1, 4.2; p = 0.03). There was no discernible difference in surgical re-intervention rates (HR = 1.4; 95% p = 0.11; CI: 0.9, 1.9).	The indigenous population's higher late death rate was linked to more difficult patients.
D. Nour et al. [15]	Retrospective observational	Indigenous	N/A	PCI and CABG	Following cardiac intervention, the indigenous population had a higher incidence of major adverse cardiac events (MACE): PCI: 30 days (1.5%), 1 year (6.1%), 5 years (28.7%), and 10 years (48.5%); CABG: 30 days (1.6%), 1 year (7.9%), 5 years (12.7%), and 10 years (34.9%).	Due to concomitant illnesses, indigenous individuals have a significant prevalence of MACE episodes after cardiac intervention.

Overall, the pooled analysis across multiple studies indicates no statistically significant difference in the odds of a 30-day mortality outcome between indigenous and non-indigenous populations undergoing these interventions. The combined odds ratio of 1.04 with a 95% confidence interval of (0.78, 1.40) suggests a neutral effect, indicating that there is no significant disparity in outcomes between the two groups.

**Figure 2:**
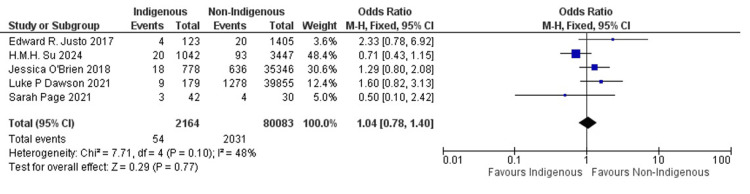
Forest plot of a meta-analysis of 30-day mortality.

Individually, when examining specific studies (Figure 2), the results vary. For instance, the study by Edward R. Justo et al. and H.M.H. Su et al. reports an odds ratio of 2.33 (0.78, 6.92) favoring indigenous patients, although this result is not statistically significant. Conversely, the study by Jessica O'Brien et al. and Luke P Dawson et al. yields odds ratios of 1.29 (0.80, 2.08) and 1.60 (0.82, 3.13), respectively, indicating a slightly higher likelihood of adverse outcomes among indigenous patients, although again not statistically significant. Sarah Page et al.'s study, on the other hand, suggests a lower odds ratio of 0.50 (0.10, 2.42), favoring non-indigenous patients, but with wide confidence intervals reflecting uncertainty due to the small sample size.

**Figure 3:**
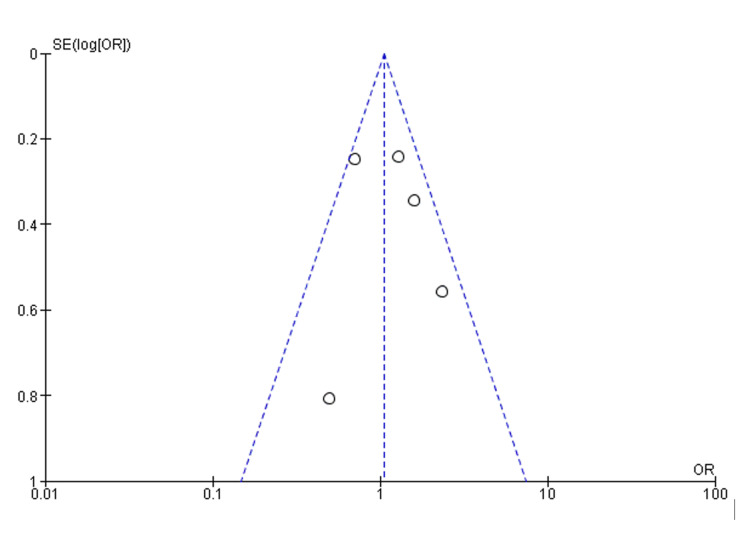
Funnel plot of 30-day mortality

Overall, the heterogeneity test indicates moderate heterogeneity across the studies (I² = 48%, p = 0.10). However, the test for overall effect shows no significant difference (Z = 0.29, p = 0.77), suggesting that while there is some variability in the individual study findings when aggregated, there is no discernible disparity in PCI and CABG outcomes between indigenous and non-indigenous Australians. A funnel plot showing the above is shown in Figure 3.

**Figure 4:**
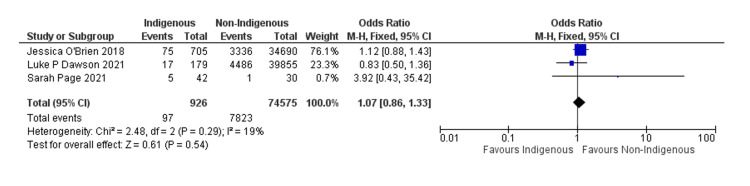
Forest plot of the meta-analysis of readmissions to the hospital

From the forest plot data (Figure 4) presented for the studies by Jessica O'Brien (2018), Luke P. Dawson (2021), and Sarah Page (2021), we can derive several findings regarding the comparison of outcomes between indigenous and non-indigenous populations about readmission to hospital.

First, when comparing the odds ratios for adverse events between indigenous and non-indigenous patients, the combined analysis yields an odds ratio of 1.07 (95% CI 0.86, 1.33). This indicates that there is no statistically significant difference in the odds of adverse events between the two groups.

Individually, the odds ratio for adverse events in the study by Jessica O'Brien (2018) is 1.12 (95% CI 0.88, 1.43), suggesting a slightly higher likelihood of adverse events among indigenous patients, although this result is not statistically significant. Similarly, the odds ratio for adverse events in the study by Luke P. Dawson (2021) is 0.83 (95% CI 0.50, 1.36), indicating a slightly lower likelihood of adverse events among indigenous patients but again not statistically significant.

On the other hand, the odds ratio for adverse events in the study by Sarah Page (2021) is 3.92 (95% CI 0.43, 35.42). However, this wide confidence interval reflects substantial uncertainty due to the small sample size, making it difficult to draw definitive conclusions from this study alone. A quality assessment of these studies is presented in Table 2.

**Table 2:** Quality assessment of the reviewed studies by the New Castle Ottawa Scale

Study	Representativeness of the exposed cohort（1）	Selection of the non-exposed cohort（1）	Ascertainment of exposure （1）	Demonstration that outcome of interest was not present at start of study（1）	Compare ability of cohorts on the basis of the design or analysis （2）	Assessment of outcome （1）	Was follow-up long enough for outcomes to occur（1）	Adequacy of follow up of cohorts（1）	Representativeness of the exposed cohort（1）
L. Dawson et al. [7]	1	1	1	-	2	1	1	1	1
Luke P Dawson et al. [5]	1	1	1	1	2	1	1	1	1
H. Su et al. [8]	1	1	1	-	2	1	1	1	1
O'Brien et al. [11]	1	-	1	-	1	1	1	1	1
Jessy A Nellipudi et al. [12]	1	-	1	-	1	1	1	1	1
Sarah Page et al. [13]	1	1	1	-	2	1	1	1	1
Kyi T.H. Win et al. [14]	1	1	1	1	2	1	1	1	1
Edward R. Justo et al. [15]	1	1	1	-	2	1	1	1	1

Overall, the heterogeneity test suggests low heterogeneity across the studies (Chi² = 2.48, df = 2, p = 0.29; I² = 19%), indicating a reasonable degree of consistency in the findings. Additionally, the test for overall effect shows no significant difference (Z = 0.61, p = 0.54), further supporting the conclusion that there is no discernible disparity in adverse event outcomes between indigenous and non-indigenous populations based on the aggregated data from these studies.

Discussion

Examining the differences in cardiovascular health outcomes between indigenous and non-indigenous Australians, a systematic review and meta-analysis of PCI and CABG results was conducted. This study offers a thorough explanation of these differences. Several recurring patterns surface from several retrospective observational studies, providing insight into the demographic traits, procedural results, and long-term death rates related to cardiac treatments among indigenous Australians. In Australia, the difference in life expectancy between indigenous and non-indigenous people is around eight years. Ischemic heart disease is thought to be responsible for 14% of the health gap, and among indigenous Australians, the death rate from the condition is twice as high as that of non-indigenous Australians (117 deaths versus 59 deaths per 100,000 people annually) [10,4]. Disparities in cardiovascular care exacerbate these greater rates of cardiovascular disease and death. Over the last 15 years, there have been significant gains in life expectancy and care inequalities; nonetheless, there are still gaps between indigenous and non-indigenous Australians in terms of access to expert reviews, angiography, and revascularization procedures [10]. First, there is a pattern that is constant across research when looking at PCI results. According to the combined findings of Dawson et al., Luke P. Dawson et al., and Su et al., indigenous patients receiving PCI exhibit different demographic characteristics than their non-indigenous counterparts [7,5,13]. They have greater rates of comorbidities, including diabetes, smoking, and renal impairment, and they are usually younger and female. Additionally, they were more likely to live in rural or regional locations as well as places of low socioeconomic status. Indigenous patients are more likely to have unfavorable outcomes, such as long-term mortality and major adverse cardiovascular events (MACE), within 30 days after surgery, even when their procedural success rates and access to adjuvant therapy are comparable. These results highlight the critical need for focused efforts to address the underlying risk factors causing indigenous Australians to have worse PCI outcomes.

The results of many retrospective observational studies comparing the outcomes of coronary artery bypass grafting (CABG) and percutaneous coronary intervention (PCI) in Australians of indigenous and non-indigenous descent show no statistically significant differences in cardiovascular health outcomes. Numerous studies, such as those conducted by Luke P. Dawson et al., H. Su et al., and L. Dawson et al., have shown no statistically significant evidence that indigenous Australians receiving these therapies are more likely than their non-indigenous counterparts to have unfavorable outcomes [11,9,12]. The underlying causes of these discrepancies are many and include cultural safety, communication barriers, socioeconomic determinants of health, and inadequate access to care. Geographical considerations have a significant influence on PCI, as shown by our data. Patients with acute coronary syndrome who live in rural or regional areas are more likely to undergo interhospital transfers and thrombolysis, which may cause delays in receiving PCI. These delays may account for Indigenous Australians' decreased left ventricular performance at the first evaluation.

The lack of difference in the symptom-to-door time between the indigenous and non-indigenous cohorts is a significant finding in this study, as it dispels a myth that may exist among healthcare providers regarding the relationship between disparities and the inaction or delayed action of indigenous Australians with ACS. Indigenous Australians had higher rates of comorbidities than other populations, including diabetes, renal impairment, and smoking, which are linked to incident coronary disease, disease progression, and mortality. These findings are in line with earlier research that linked this disparity to a lower socioeconomic status and social determinants of health [13,14]. Indigenous Australians had a lower percentage of PCI for non-ACS reasons, which may be related to limited access and challenges getting to non-invasive testing centers for the diagnosis of stable coronary syndromes. Similar difficulties arise in post-PCI care, with poorer overall follow-up rates that are in line with earlier research [15].

Research on isolated CABG surgeries (O'Brien et al., Jessy A. Nellipudi et al.) revealed that, compared to non-indigenous patients, indigenous patients presented younger, had more comorbidities, and had a greater risk of long-term death [11,12]. In a similar vein, Sarah Page et al. discovered that Indigenous patients who arrived at the emergency room complaining of chest discomfort had a greater prevalence of serious adverse cardiac events and more cardiovascular risk factors [11]. 

Additionally, studies by Edward R. Justo et al. and Kyi T.H. Win et al. examined the outcomes of heart surgery in indigenous patients and discovered that although death rates were similar to those of non-indigenous patients, indigenous patients had lower long-term survival rates and higher rates of return to the operating room [14,15]. These findings were attributed to higher patient complexity and socioeconomic deprivation. Furthermore, a greater frequency of significant adverse cardiac events after cardiac intervention was found by D. Nour et al. in indigenous patients, underscoring the need for better care of comorbid diseases and focused preventative initiatives [16].

Research by Luke P. Dawson et al. and Sarah Page et al. brought attention to the socioeconomic and regional differences that indigenous Australians experience. Indigenous patients were more likely to live in rural or regional locations, as well as in regions with lower socioeconomic status, which may make it more difficult for them to get timely and high-quality medical treatment [5,13]. The quantity and quality of information about the cardiovascular health requirements of indigenous people living in both urban and rural environments need to be increased immediately. We brought attention to the significant variations in indigenous health outcomes after PCI in this research.

In Australia, improving the health condition of Indigenous people has long been an aim, yet the disparity in health outcomes is still unacceptably large. Due to their emphasis on differences in access to diagnostics, angiography, and revascularization, earlier studies have added to the deficit narrative in indigenous health research [15]. Research on the health consequences for indigenous people after revascularization is scarce. Our research, which adopts a solutions-focused methodology, emphasizes the need for all health providers, communities, and policymakers to prioritize follow-up after PCI as a means of enhancing health outcomes for indigenous Australians. Enhancing primary care accessibility, non-invasive cardiac testing, and specialist follow-up (e.g., via telemedicine) as well as optimizing care pathways for outlying and regional ACS patients to shorten treatment wait times might prove to be very advantageous. The results have special significance for the Aboriginal population of Victoria, who have lived in Australia for thousands of years and are losing their next generation of elders due to early-onset coronary artery disease and associated repercussions. Therefore, enhancing treatment routes is crucial for maintaining indigenous cultural knowledge and customs in addition to enhancing health outcomes.

Jessy A. Nellipudi et al. and O'Brien et al. concentrated particularly on the results of individual CABG surgeries. The study revealed that while the short-term death rates of Indigenous and non-Indigenous patients were comparable, the long-term mortality risk was notably greater for the former group. This underscores the need to implement customized preventive methods to enhance outcomes after coronary artery bypass grafting [1,12].

There are a few limitations to consider, even though this systematic review and meta-analysis provide insightful information on the differences in PCI and CABG results between indigenous and non-indigenous Australians. First, the majority of the included studies were retrospective observational studies, which are biased by nature due to selection bias and confounding factors, among other issues. Furthermore, the results' generalizability may be restricted by the variations in research designs, patient demographics, and outcome measures across the investigations. Furthermore, the accuracy and dependability of the estimations may be impacted by the relatively small sample sizes in certain research studies, especially those that concentrate on indigenous groups. Moreover, a significant number of studies failed to explicitly address socioeconomic determinants of health, communication obstacles, or cultural factors-all of which have a significant impact on healthcare inequalities and outcomes among indigenous communities. Lastly, even though attempts were made to include research done within the last 10 years, publication bias or search techniques may have left out pertinent studies.

Future studies should focus on overcoming these constraints and clarifying the variables behind the differences in PCI and CABG results between Australians of indigenous and non-indigenous descent. Prospective studies that allow for the investigation of causal linkages and have bigger sample sizes and longer follow-up periods would provide more reliable results. Furthermore, research using qualitative methodologies may shed light on the social and cultural factors of health that affect healthcare outcomes in indigenous communities. In addition, it is important to conduct thorough assessments of interventions that target socioeconomic determinants of health, culturally competent care delivery, and enhanced access to healthcare to ascertain their efficacy in mitigating healthcare inequalities. To guarantee that research goals and procedures are relevant to the communities under study and suitable from a cultural standpoint, it is imperative that continuous efforts be made to develop research capacity within indigenous communities and include indigenous stakeholders in the research process.

## Conclusions

The findings of this systematic review and meta-analysis demonstrate no statistically significant differences in PCI and CABG outcomes between Australians of indigenous and non-indigenous descent, with the former group generally enduring greater rates of adverse cardiovascular events. Notwithstanding the progress made in the field of cardiovascular care, there are still gaps in the treatment of Indigenous Australians due to issues with financial status, cultural safety, and access to care. Multifaceted initiatives aiming at enhancing care access, culturally competent healthcare delivery, and addressing socioeconomic determinants of health will be necessary to overcome these discrepancies. By tackling these fundamental causes, we may endeavor to get fair cardiovascular results for indigenous Australians and lessen the prevalence of cardiovascular illness in these groups.
